# Application of Digital Twins in multiple fields

**DOI:** 10.1007/s11042-022-12536-5

**Published:** 2022-02-16

**Authors:** Jinkang Guo, Zhihan Lv

**Affiliations:** 1 School of Data Science and Software Engineering, Qingdao University, Qingdao, 266071 China; 2 Department of Game Design, Faculty of Arts, Uppsala University, Uppsala, Sweden

**Keywords:** Digital Twins, Virtual reality, Industry, Personalized medicine, Aerospace, Logistics

## Abstract

With the development of science and technology, the high-tech industry is developing rapidly, and various new-age technologies continue to appear, and Digital Twins (DT) is one of them. As a brand-new interactive technology, DT technology can handle the interaction between the real world and the virtual world well. It has become a hot spot in the academic circles of all countries in the world. DT have developed rapidly in recent years result from centrality, integrity and dynamics. It is integrated with other technologies and has been applied in many fields, such as smart factory in industrial production, digital model of life in medical field, construction of smart city, security guarantee in aerospace field, immersive shopping in commercial field and so on. The introduction of DT is mostly a summary of concepts, and few practical applications of Digital Twins are introduced. The purpose of this paper is to enable people to understand the application status of DT technology. At the same time, the introduction of core technologies related to DT is interspersed in the application introduction. Finally, combined with the current development status of DT, predict the future development trend of DT and make a summary.

## Introduction

### DT development history

**Fig. 1:**
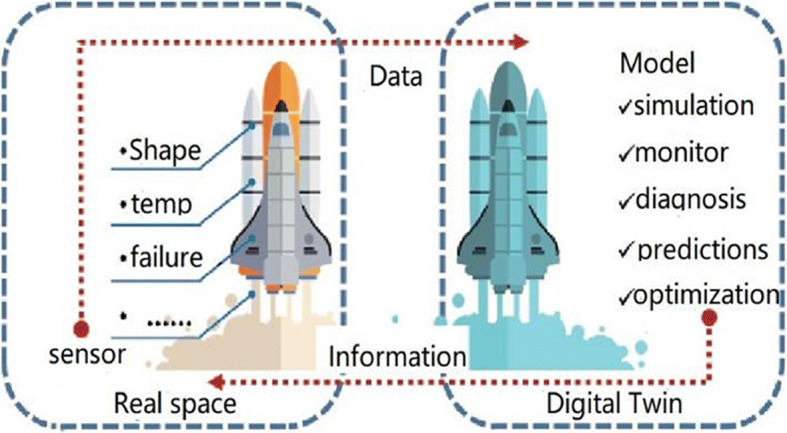
Information mirroring model

The concept of digital twins first appeared in the US Apollo 13 rescue mission in 1970. After the spacecraft was launched into space, the oxygen tank suddenly exploded and the situation was critical. In order to save the astronauts, the National Aeronautics and Space Administration (NASA) used Apollo 13’s digital model to simulate it. After many attempts, it worked out a solution and sent it to the astronauts in the spacecraft. This incident can be said to be the earliest practice of applying digital twins. The prototype of DT technology was the first to present the PLM model in a slide produced by Michael Grieves in 2002 to give a presentation to industry at the University of Michigan for the establishment of the PLM (Product Lifecycle Management) Center [56]. Both the concepts of real and virtual space are mentioned in the model, which already have all the elements of a DT. Then, in 2006, the information mirroring model has been extended. The model has a preliminary idea to deal with the reality and virtual existence in the digital twin, but it does not propose how the two interact, and it does not indicate which technology to use to achieve it (Fig. 1).

In 2010, the digital twin was formally proposed by John Wicks in the 2010 NASA roadmap report, marking the official birth of the DT. In 2012, NASA began using the term “Digital Twins” in its technology roadmap.Since 2014, some well-known companies such as Siemens, Dassault, PTC, ESI and ANSYS have been using the term “Digital Twins” in their marketing campaigns. And in the DT of the technical construction, conceptual connotation has done a lot of in-depth research and expansion.

### Definition

DT is a technology that combines multiple fields [74]. DT currently lack a unified definition, which is still in constant development and evolution. The definitions of data twin in different units and stages are listed as follows:

- 2012 NASA defined DT

DT is a comprehensive multi-physical, multi-scale, probabilistic simulation system for vehicles or systems. It uses the best physical model to describe the historical use of equipment to reflect the life of its corresponding physical equipment [28].

- 2017 DT defined by the Defense Procurement University

The integrated multiphysics, multiscale, probability simulation, using the best available models, sensor information, and input data to mirror and predict the life/activity/performance of the ircorresponding physical Twin, enabled by Digital Thread [73].

- 2019 Stark Damerau defined DT

A DT is a digital representation that contains the feature description of its selected object or its product and service system, and obtains the attributes, conditions and behaviors of the object through models, information and data in a single or even multiple life cycle stages. [63].

- 2020 DT defined in a white paper published by the China Institute of Electronic Technology Standardization

DT refers to making full use of data such as physical model, sensor update, operation history, and integrating multi-disciplinary, multi-physical, multi-scale, and multi-probability simulation process to complete the mapping in the virtual space, thereby reflecting the full life of the corresponding physical equipment Cycle process [24].

In summary, the DT is a multi-faceted definition. Be able to include all high-tech fields. Fuller suggests that artificial intelligence is becoming an integral part of DT, exploring where AI algorithms can beapplied, which is also a future direction [26].

### The characteristics of the DT

The DT constructs a high-fidelity model consistent with the physical world in the virtual space in the form of digitization. Through the uninterrupted closed-loop information interaction feedback and data fusion with the physical world, the behavior of objects in the physical world can be simulated. As can be seen from the definition of DT, DT have the following characteristics:

1. *Concentration*. All data during the life cycle of a physical system is stored on a digital mainline for centralized and unified management, making the two-way transfer of data more efficient.
2. *Integrity*. For complex systems, DT integrate all subsyscies, which is the basis for high-precision modeling, while real-time monitoring of data can further enrich and enhance the model, enabling the model to contain all the knowledge of the system.
3. *Dynamic*. Sensor data describing the physical system environment or state can be used for dynamic model updates, updated models can dynamically guide the actual operation, and real-time interaction between physical systems and digital models enables models to grow and evolve throughout their life cycle.

The characteristics of DT allow it to change the execution process of traditional projects. Traditional project design processes typically start with requirements, then implement, test, and then iterate through the process, often requiring interaction between different departments. However, there are always problems with information interaction between different departments, resulting in inefficient design. DT can manage all kinds of information in a unified way, and different departments can access and add data from DT at any time, allowing departments to work in parallel. Traditional system design needs to manufacture physical equipment first, and then verify the system. Virtual simulation of DT allows virtual testing to be performed before physical devices are manufactured. The traditional modeling approach is to predict the environment and load that may be experienced in the future based on historical data, and to use this model to guide the production of subsequent products. It is inevitable that errors will occur in the actual production, and it is difficult to simulate the real environment during the design phase. In the whole life cycle of a physical system, DT uses real-time monitoring data to drive dynamic updating of the model, so that the model can not only reflect the state of the device, but also track the changing system environment in a complex environment.

It is DT that have these advantages, so they can be applied to a variety of real-world scenarios. This paper will introduce the application of DT in industrial production, health care, smart cities, aerospace and business, as well as the current development status quo, and make some predictions for their future development, pointing out their future development trends.

## The application of DT in industrial production

**Fig. 2:**
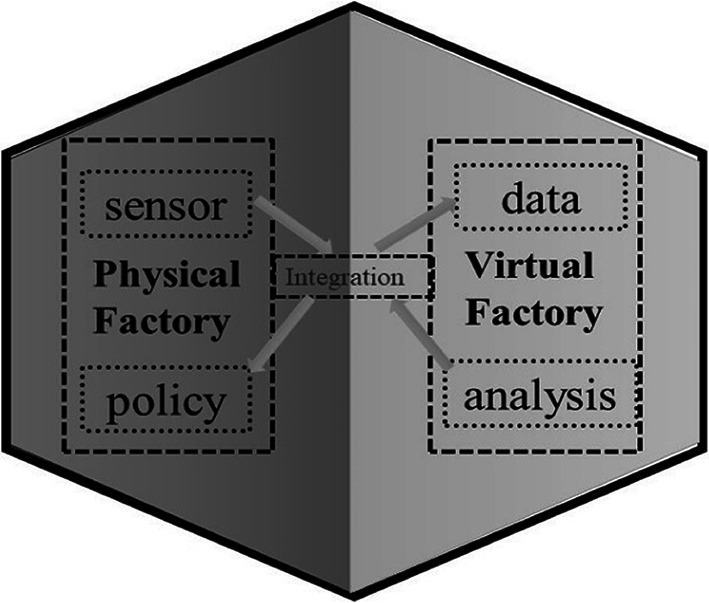
The application of DT in smart factories

Modern industry has entered the development process of all-round digitalization. In production systems aimed at improving productivity, product quality and product performance, modern industry is increasingly dependent on digital design and production processes. In the process of transitioning to Industry 4.0, one of the problems that needs to be solved is to build a communication channel between the physical world and the digital world. This is the role of Digital Twinss [49]. Digital technology not only provides an opportunity for accelerated innovation in industrial production, but also greatly changes the traditional model of industrial system and injects new impetus into the traditional production system. Today, under the co-ordination of the industrial Internet, industrial intelligence is gradually taking shape, and DT are the key to leading industrial intelligence. At present, this technology is mainly used in “high value” areas, such as rocket manufacturing, oil and gas extraction systems. According to Gartner’s technology trends analysis, DT technology will mature in the next 5 to 8 years. With the popularity of 5G and industrial IoT platforms, global DT technology is bound to be used on a large scale. Now, industry is entering the era of Industry 4.0, and DT will play a very important role in the transition from Industry 3.0 to Industry 4.0. The application of DT in Industry 4.0 is a hot topic of research and an urgent problem to be solved [69]. Urban August, Senior Vice President, Siemens, says DT is an essential technology for the transition from traditional to digital enterprises [5] (Fig. 2).

**Fig. 3:**
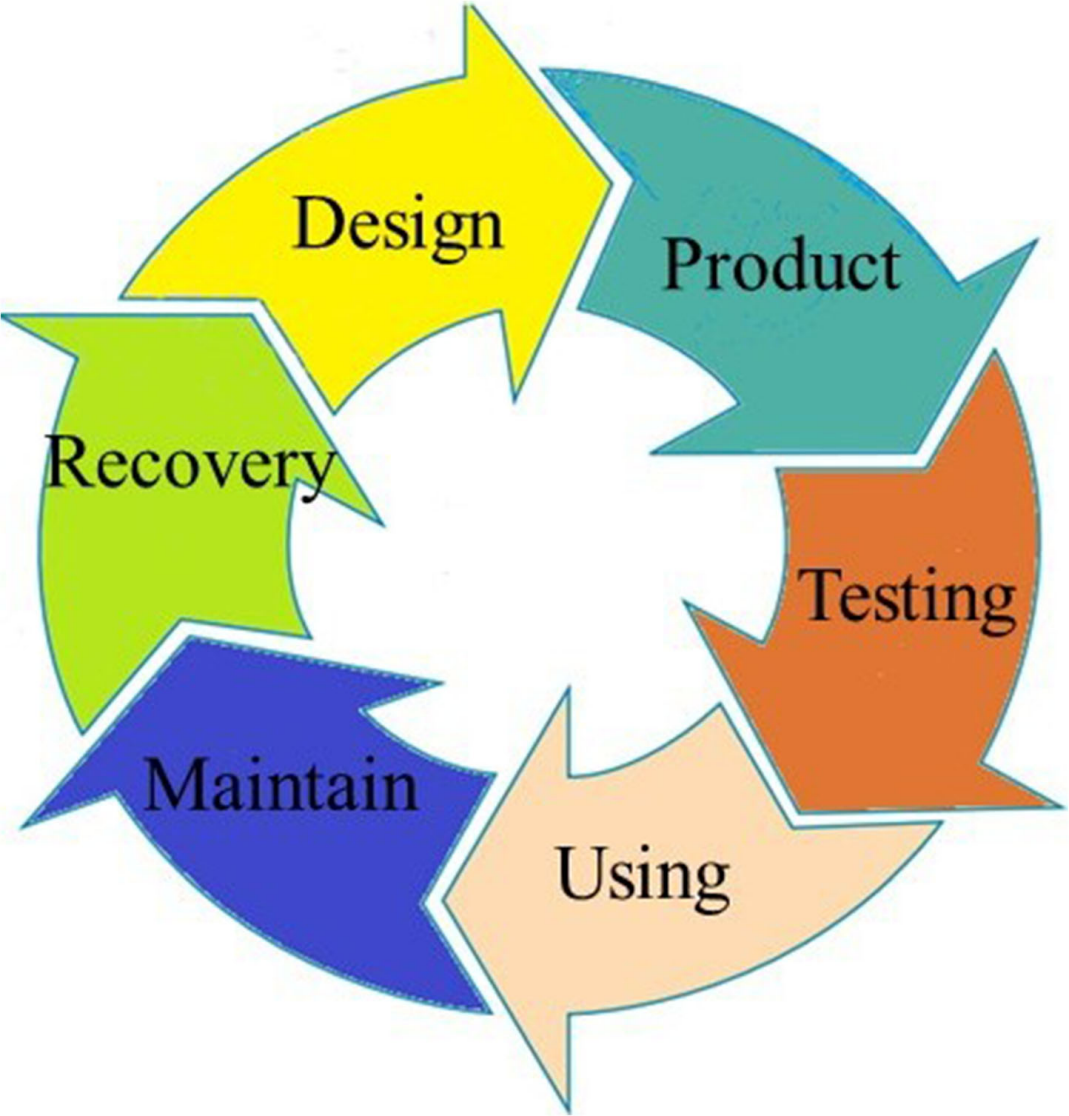
DT go deep into all areas of product manufacturing

As shown in Fig. 3, the smart factory based on the DT plays an important role in every link in the life cycle of product production.

1. For product designers, they can rely on DT to carry out product reliability simulation and analysis, and then carry out design improvements and improve product usability.
2. For manufacturers and validates, reliable evaluation can be carried out using DT and combining products in kind.
3. For the maintainer, the DT can rely on the product’s security analysis and predictive maintenance and other work. When the product is scrapped, it can rely on DT to carry out the residual life analysis and re-manufacturing evaluation of each system of the product, which will help the design of the next generation of products.
4. For users, digital twins can be used to predict product availability and have a more comprehensive understanding of the product. At the same time, the system can be used more conveniently in use.

Regarding the application of DT in industry, many scholars have also done relevant research. Thomas [69] suggest that DT in Industry 4.0 can automate data acquisition and data processing, and introduce a multi-mode data acquisition method. Jiapeng Guo [30] proposed a modular DT factory design, using a modular idea to design the factory. The idea of a modular approach is to pre-build digital modules that correspond to physical entities and model only important parts of the plant. When the production demand of the factory changes, the modular DT project only needs to adjust some modules to achieve the corresponding demand, which is very flexible. Shvedenk [62] proposes a digital dual projection interaction method based on the decomposition of physical system objects, using a common-line structure to ensure the joint use of data streams formed by DT projections. Chaiwat Assawaarayakulp [4] proposes another way to develop Industry 4.0 by proposing a DT that can be self-developed and built, combined with the structure of the pyramids of an automation plant. Using DT as a channel for communicating the layers of the pyramid allows plant automation without changing the structure of the plant. Siemens uses DT in its plants to debug equipment, and in virtual spaces it is very efficient to debug complex equipment [66]. Simulating complex systems with DT allows as many key employees as possible to participate in project development and deployment, as well as making it easier to configure and adapt devices, increasing deployment flexibility. In terms of modeling, the Zhang, H’s steam [82] proposed a CCPS information modeling approach based on AutomationML. It provides a safe and efficient data modeling method for the factory. The role of AutomationML is to encapsulate various manufacturing and industry-related services and definitions, and then integrate DT into cyber-physical production system (CPPS) to provide services for factories. CCPS is a multi-dimensional complex system that integrates computing, network and physical environments, and is used for complex system management.

**Fig. 4:**
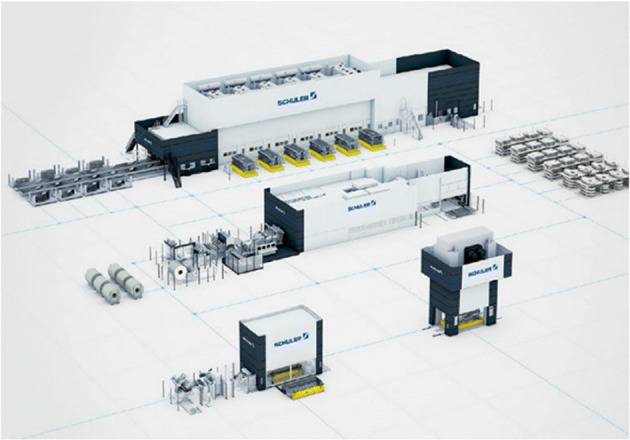
This image is excerpted from the concept map of Schuler’s Smart Factory [59]

Schuler has started adding DT to its actual production [59]. Simon Scherenbacher, Schuler’s vice president, has come up with a solution for deploying a DT plant at Schuler, with initial results. Accurately predict possible downtime with Smart Press Shop and prevent downtime, greatly improve factory production efficiency. Since 2016, the machines in the Schuler press production line have been equipped with sensors to collect data from the machine tools. This means that they have been able to monitor the system status and operation process in detail. In addition, the intelligent diagnosis function realized by the machine learning algorithm can conduct retrospective analysis to determine the cause of the failure when the production line fails(Fig. 4).

It can be seen from the current research that in the future, DT will be the core technology of future smart factories. DT technology can optimize the original model after application, and can innovate the product service model to fully meet the needs of consumers for DT data resources. Nevertheless, there is also a significant gap in the degree of development of DT in different countries [72]. The popularity of Digital Twinss in the world industry is still a long time away. In addition, the industrial Internet requires a high level of network reliability andreal-timeper formance [49]. However, in the current scenario, very little is proposed on how to ensure the reliability of the digital model. Here, I propose a plan, which is to apply the blockchain to the DT. Use the cryptographic characteristics of the blockchain to ensure the data immutability of the DT program.

## The application of DT in the medical field

Modern medicine includes not only the treatment of patients, but also health management, disease prevention and health recovery. And in addition to physical treatment, but also psychological treatment. With the popularity of the Internet, people’s demand for medical services is shifting from offline mode to online-based, offline-assisted new models. And the current medical field for the elderly health services, chronic diseases and sub-health groups of the service is relatively lacking. During the new crown epidemic, people living in homes began to realize that medical facilities in the home were lacking and that there was a lack of effective ways to provide residents with health guidance and treatment options from doctors.

The psychological treatment of patients is usually realized by virtual reality technology. The current applications of DT are mostly used in physical therapy. As a multi-aspect fusion technique, DT can construct digital models of living entities. Life is a complex “system”, which requires the management system to have powerful computing capabilities. On this basis, it is best to be able to manage the system in the same way as a biological brain. Shuangming Yang [76] proposed the use of multi-chamber simulation to study large-scale biological nervous systems, and proposed a multi-optical neuron architecture (CMN). At the same time, Yang [77] also proposed a biological-inspired cognitive supercomputing system (BiCoSS) to further explore the neural mechanisms behind the human brain. This provides theoretical support for the application of Digital Twinss in medical treatment. DT, as a form of multimedia, can visually display various data of the human body. There are currently experiments that simulate the spread of bacteria in corpses after death [60]. By building digital models of life through DT, the whole life can be controlled, providing a new way for people to achieve disease treatment, health monitoring, motion management.

### Treatment of diseases

The main medical applications of DT today are mainly in the field of complementary therapy. For example, as an immersive interface for medical software by virtual doctors or nurses [23]. Siemens’s Healthineer DT of the human heart provides patients with 3D images of their organs and simulates their physiological condition, enabling ai-based personalized heart therapy [45].

With DT, the patient’s postoperative status can be planned in advance. For example, Post-hepatectomy liver failure (PHLF) is the leading cause of postoperative death in liver patients, and treatment options are adjusted by DT simulation surgery to predict the risk of developing portal hypertension (PHT) [29]. In cardiovascular therapy, researchers have developed a semi-active DT that detects Carotid artery stenosis from head vibrations [38]. Simulate the patient’s response to treatment so that potential complications can be predicted. In order to record the patient’s treatment results and recovery, a dynamic system has been created that describes the patient’s status and can reflect the patient’s status at anytime [36]. In terms of remote surgery, the reliability and real-time communication should be ensured. For this purpose, the Heikki Laaki’s team [33] developed a DT system. This system simulates the communication model of remote surgical task by DT for reliable communication to analyze communication needs in mission-critical applications (such as remote surgery supported by mobile networks). Therefore, the network communication ability of actual remote surgery can be guaranteed. Based on this study, the future application of DT technology in surgical medicine is derived.

**Fig. 5:**
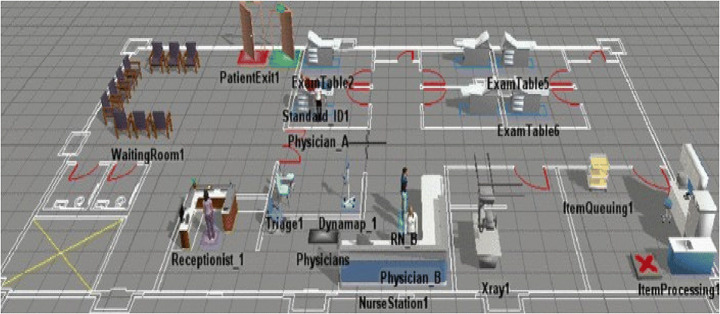
Karakra’s team uses Flexsim to simulate patient movement paths [33]

Hospitals can also be seen as a small “factory” that contains a variety of services that need to find a balance between medical resources and patients. Yet, hospitals are currently facing changing medical needs. This requires hospitals to constantly add new services as they demand, and to make those services more efficient. For the management of an enterprise, Barat [8] has proposed building an enterprise’s DT from a DT to evaluate the effectiveness of a series of measures in the enterprise. By building the hospital’s DT, Karakra [33] controls the hospital’s resource utilization, adjusts the current hospital’s service plan, effectively improves the utilization rate of medical resources, and plans for the hospital’s future development. After deploying the model in the hospital, the waiting time for patients was reduced by an average of 20-30 min. And improve the resource utilization rate of the hospital (Fig. 5).

**Fig. 6:**
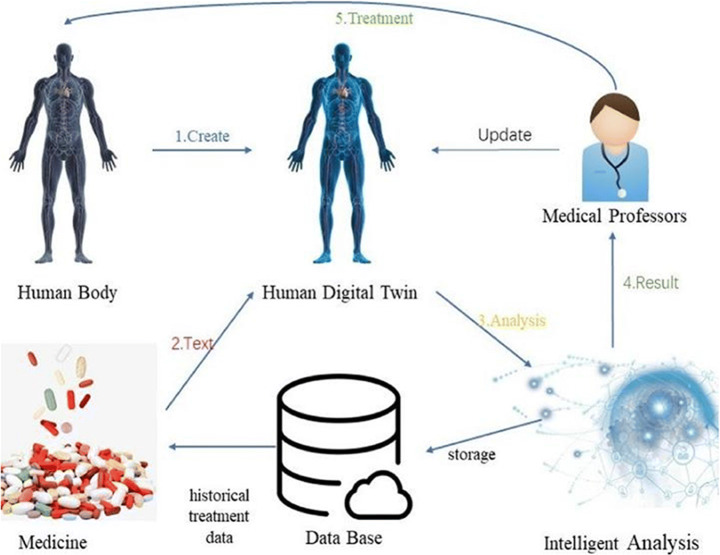
Precision medical process

DT can also enable patient precision medicine in the future, or personalized medicine, i.e. different treatment options for different patients with the same condition. Precision medicine maximizes the use of medical resources, uses the least amount of medication for the best treatment, and reduces the side effects of the drug on patients. Personalized medicine can be easily achieved through DT. First, collect the patient’s body data to construct its digital model. Then, the common drugs for the disease will be found from the database to test the patient’s digital twin mirror image. The attending doctor obtains the results of intelligent analysis and updates the twin mirror of the patient based on experience. This process is repeated continuously until the best medicine is found and stored in the database. Finally, the doctor treats the patient (Fig. 6).

DT can build digital copies of all molecules associated with disease mechanisms in a single patient and computer-process DT with tens of millions of drugs to determine the best drug for treatment [2, 10]. Precision medicine leverages the intelligence benefits of big data by connecting personal data to society. Therefore, precision medicine encourages the prevention, containment or timely detection of diseases according to the patient’s personal situation. The advent of precision healthcare means seeing a digital transformation of technology-driven healthcare that will enable personalized prognos processing through innovativeand targeted medical approaches [11]. For example, studies related to obstructive sleep apnea(OSA) have found that there are many causes of OSA, often due to a combination of factors. Among them, craniofacial malformation or syndrome, as well as anatomic and geometric changes in the upper respiratory tract, as well as weight gain and age increase are the main causes of the disease. Using the DT model of patients to pre evaluate the therapeutic effect of treatment procedures can significantly reduce the time required for diagnosis and treatment. And according to the status of different patients, provide specific treatment. By adding age-related biological parameters to the “Digital Twins” model and integrating the data, we can predict the occurrence of OSA and carry out preventive intervention [39].

### Health management

In recent years, the number of health institutions, the number of medical personnel, the assets of medical institutions, the per capita hospitalization costs and the number of insured people have shown an increasing trend year by year [41]. At present, most of the medical resources are available for treatment, and with the increase in the population, the problem of inadequate medical resources will continue to be exacerbated. Through disease prevention, health management and disease recovery, the frequency of disease will be reduced and medical stress will be alleviated. However, there is a serious shortage of medical facilities and experts in these areas, which is difficult to reach in society. DT provide a viable way to monitor people’s physical condition in real time and provide strong support in people’s cloud health care services. Among them, the proposed DT health care (DTH) and health 4.0 point out a new direction for future medical development. DTH is a new type of medical concept used in medical activities or medical systems. It uses DT technology and multi-science, multi-physics and multi-scale models to provide fast, accurate and efficient medical services [39]. The realization of DTH can provide patients with comprehensive health monitoring. Health 4.0 is defined as the transition from passive to active health care, building DT from parameters such as physical health, lifestyle, mental health, socioeconomic health (income, housing), environmental health (family, work), culture and religion, and thus determining people’s health [7]. Health 4.0 as a measure of people’s health in the future, provides a direction for the future development of the healthcare industry.

There are currently many DT used in health management. Bagaria [29] receives people’s physical indicators through sensors such as smartwatches, and then builds digital models to manage people’s mental health. He found that changes in people’s emotions can change the state of some organs, which in turn causes changes in the physiological state of the human body, which allows people’s emotions to be monitored. Afterwards, data analysis is used to find out the source of stress, and intervene in user behavior to reduce user stress. Doctor Li [39] proposes a cloud healthcare system framework based on the DT Healthcare System. This is a scalable framework in a cloud environment. The system combines cloud computing and DTH to ensure that users can quickly provide high-quality services. The system will allow user data to be stored in the cloud, taking advantage of cloud-based privacy mechanisms to secure your data. Organizing data by hashing can speed up data search [39, 64].

Health management for chronic patients can effectively prevent the deterioration of the disease and improve the condition to a certain extent. Vaskovsky [70] analyzes the dietary records of diabetics using DT technology and develops a diet plan based on the patient’s condition and personal preferences.

### Sports planning

Exercise is an important way for people to keep healthy, but it is difficult to make healthy exercise plans according to their own situation. Especially for athletes, how to develop training plans to improve performance is the most important concern of every athlete. DT can monitor the state of the body in real time by embedding sensors in wearable devices, obtain physical information from people in operation, store measurements as historical data, perform further data analysis, and provide the ability to make reliable predictions. According to the physical condition of the movement, the movement plan is adjusted in real time. In addition, Digital Twins can also develop personalized exercise plans for ordinary people [56].

**Fig. 7:**
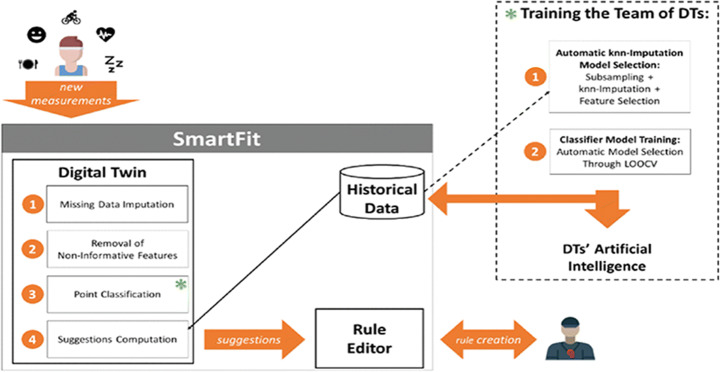
Architecture of the Smart Fit [9]

At present, the Smart Fit system launched by the Barricelli [9] team can help coaches of professional sports teams to monitor and analyze the health status of athletes. Its architecture diagram is shown in Fig. 7. The system stores data such as the number of calories consumed and burned or sleep time, and describes the athletes’ Behaviors (e.g. food income, activity, sleep) over several consecutive days. Through this system, the coach can observe the athlete’s status in real time and adjust the training plan. Smart Fit can predict the physical condition of the athletes and make suggestions for revisions to the exercise plan.

## The applications of DT in smart cities

We are currently in a new era of urbanization, with the world’s urban population accounting for 50% of the total population, and the scale and pace of urbanization accelerating gradually, which is known as the second wave of urbanization. At the same time, urbanization has been growing faster than in the past since the beginning of the third millennium, an era in which information technology is used in all areas of urban life and can be called the third wave of urbanization [22]. According to United Nations estimates, the world’s urban population is expected to double by 2050, when two-thirds of the population will live in cities. The acceleration of urbanization has become a major burden on many urban systems [47]. As the world’s urbanization continues to accelerate, the structure of cities has become more and more complex. With the emergence of various large cities, it is increasingly difficult to manage cities. It is in this context that the popularization of smart cities is becoming more and more necessary. With its inherent advantages of facilitating data transparency and convenient management, smart cities are the best development direction for future cities. The core idea of a smart city is to build a bridge between human capital and social capital. Through the combination of the two, a greater and more sustainable economic development and a higher quality of life for citizens [67]. The intelligentization of cities will become the trend of future development.

The emergence of DT provides a shortcut for the development of smart cities, and the construction of smart cities echoes the characteristics of DT. Smart cities have the characteristics of multidimensional perception, multidimensional data, and multidimensional intelligence. Traditional two-dimensional displays can no longer meet the display requirements and interaction requirements of smart cities. DT have the characteristics of integrating multiple disciplines, multiple scales, and multiple probabilities, which can well meet the virtual mapping of smart cities to the real world. DT have a natural advantage in the construction of smart cities.

### What is smart city?

The concept of a smart city dates back to 1994, when the city of Amsterdam was the first city to commit to creating and integrating the concept of a virtual digital city [3]. Limited to the level of science and technology at that time, smart cities were not vigorously promoted in that year. After that, smart cities have been developing in twists and turns until the emergence of digital twins, which provides a new way for the development of smart cities. Ivano [32] proposes a definition of a DT for a smart city: a city’s DT system is a system in which multiple DT are connected to each other. Data from different sources build different DT, such as data on traffic flow and people flow can monitor and predict the flow of people, various sensors can obtain the temperature and humidity of the city and other parameters, can predict the city’s environmental situation, urban camera image data can reflect the city’s environmental state, build a city 3D model. He points out that smart cities should include functions such as monitoring the state of the urban environment, rapid emergency response, efficiency assessment of solutions, identifying sources of potential risks, urban development forecasting, and so on.

### The application of DT in smart cities

The application of DT in smart cities is multi-esmic, the most prominent of which is the application of DT in smart city visualization. Before DT, the most common was to use the city’s 3D model for data visualization. 3D models as a very intuitive multimedia display tool, widely used in property display and land planning, such models only consider the appearance and shape. The latest DT will turn 3D models into a source of information related to urban landscapes and urban environments, and become the basis for managing smart cities. In the context of smart cities, using DT brings not only more intuitive urban monitoring, but more importantly, the creation of models to predict the future of cities. For example, if you increase the flow of a road, what is the impact on noise and air quality in the area? DT require a large number of observations that define causation and mathematical models between multiple factors. A large number of machine learning algorithms are used in the construction of simulation models for prediction. Energy Atlas’s [55] Kattohukka service can create dynamic city models. Obtain the needs of citizens during urban planning and predict the income of future real estate.

In the area of smart city management, smart city also has certain results. In 2018, Foth [12] discussed the four-phase revolution in the relationship between urban government and citizens. It is pointed out that the best relationship between the citizens and the government in the future is the collaborator and the co-creator.The participation of citizens in smart cities will be more conducive to the construction of cities. However, traditional forms of public participation, such as public consultation, questionnaires, public meetings, etc., are inefficient and difficult to ensure that decision-making is fair and reasonable. He proposed the concept of digital citizen participation for citizens to participate in the management and construction of smart cities. Yeji [79] also proposes a “self-organizing model” to manage cities, i.e. to construct digital models of cities through DT, involving every citizen in the construction of cities. In 2020, Svítek [65] proposed a similar approach to the management of smart cities, by combining artificial intelligence with humans to balance conflicts of interest in cities and to involve every citizen in the construction of cities, with an emphasis on involving citizens in the construction of cities of the future. Dubai’s “Happiness Agenda” is a benign case of a smart city that involves citizens in urban construction, and Dubai calls it one of the “happiest” places to live, defining the “happiness” of citizens through various parameters, and rationally allocating urban resources through big data analysis, thereby improving the “Happiness Index” of the city as a whole [80].

**Fig. 8:**
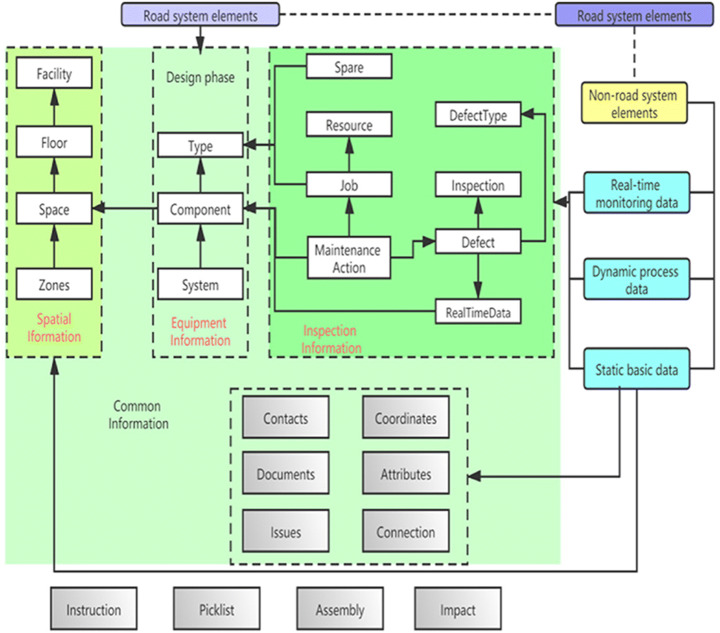
COBieber for road maintenance [27]

DT are also of great help to the construction of urban public facilities. To meet the future transportation needs of smart cities, Gang Yu [27] and his team devised a solution (ICT Artifact)that follows the Framework of Design Scientific Research (DSR) for intelligent urban road operations, proposed methods for organizing, collecting, and integrating spatial data for the entire life cycle of roads, and defined the functional requirements for smart city roads in the future. made a COBieber model for road maintenance. The difference between this model and other models is that it adopts the COBie standard, that is, not by ID but by composition to manage the equipment in the road. In the design stage, the system information is integrated into the composition set, and different devices are distinguished by the device type in the set. Afterwards, it is a good idea to obtain and maintain spatial information in units of collections, which facilitates the management of road equipment. De Luca [16] also suggested that Notre Dame could be restored through DT. Reflects the application of DT in engineering buildings (Fig. 8).

In terms of citizen security, Newark, NJ City, USA, provides intelligent solutions through a data-based smart city platform that analyzes crime reports, criminal history of suspects, conducts crime risk analysis in specific areas, combats criminal behavior, and maintains urban security [17]. In terms of personal health, IEEE proposed the ISO / IEEE 11,073 standardized DT framework architecture for health and well-being. The framework covers a series of processes of collecting data from personal health care devices, analyzing the data and giving feedback to users. When the system is used in the city level environment, it can reflect the overall health of the citizens and improve the city’s medical services [37].

### Smart Cities uses technology related to DT

In the development of smart cities, the first thing is to model existing cities. Because cities are too complex, building DT models of cities is a difficult task. To this end, many researchers have done a lot of research on this issue. Villanueva [71] combined LiDAR and Openstreetmap project data to automatically generate building models and surrounding environment models. Fan Xue [75] uses Light Detection And Langing (LiDAR) point clouds for urban object modeling, and uses format tower design principles to create city objects in the LiDAR point cloud autonomously through unsergencyed methods, greatly improving the efficiency of urban modeling.

To build smart cities with DT, a highly digital city is needed as a stepping stone [19]. The Internet of Things in Cities provides a data base for DT deployments of smart cities. In order to manage the large number of Internet of Things in smart cities and generate reliable DT, the Gabriel Tamura’s team [54] proposed using GEMINIS to apply IOT to Digital Twinss, so that Digital Twinss can better cope with the dynamics and uncertainties of the physical world, and ensure the consistency of behavior between DT models and physical entities. On the basis of the IOT with cloud computing, large-scale economy, application standardization, and software and service solutions can be realized, accelerating the learning curve of smart city operations [14].

When using DT for prediction, many artificial intelligence algorithms are used to build multiple data models. Through data mining to find out the influence between the various factors, mainly using apriori, FP-Growth and Eclat algorithm, through the neural network model combined with data mining model to predict the value of factors and indicators, through the data mining model to obtain the degree of influence of different factors to train neural networks, to obtain correct prediction results. For example, Xu Du’s [20] team used Apriori to evaluate the characteristic data of smart cities. Xiaodong Yang’s [78] team combines Apriori and FP-growth algorithms to provide optimization solutions in the construction of smart cities.

### The outlook for the future

DT, as an integral part of smart cities, show us the prototype of smart cities of the future, but there are still many questions about the seamless interaction of smart cities as digital models and physical cities. The current development of DT smart cities is currently stuck in GIS and BIM fusion, used for web and mobile plat forms for urban demonstration [15]. For the future development of smart cities, it is mainly to look at the city’s control over human resources. Ricardo Matheus [42] and others compared the development of two smart cities and found that smart cities focused on ambidexterity were more likely to succeed. Ambidexterity refers to the development of resources and innovation capabilities. And they found that the most important thing to improve this ability is to acquire human resources. This year has been accompanied by the explosion of COVID-19, which has increased the demand for telecommuting and reduced costs for companies by making it unnecessary for companies to rent offices. The main problem with remote work at present is the large gap between the working environment at home and the company. The construction of smart cities may be a way to achieve telecommuting in the future.

## The applications of DT in the field of aerospace

From an aerospace perspective, DT can be defined as an integrated tool for very advanced reality simulation settings [48]. In 2010, NASA released 《Nasa’s Roadmap for Space Technology》, which sets out its goal of achieving NASA’s DT by 2027 [61]. The report also gives four scenarios for DT technology:

1. Used for the “test flight” of the aircraft before launch. Analyze the impact of different task parameters, and research and verify the corresponding processing strategies for various abnormal phenomena;
2. Mirror the actual flight of the aircraft. Real-time monitoring of load, temperature and structural damage status, reflecting real flight conditions;
3. When the sensor indicates the degradation of the structural performance state, it diagnoses the cause of the abnormality and analyzes the post-failure response measures;
4. Simulate the health of some parts after they fail to determine whether design improvements are needed to avoid unnecessary modifications and adjustments.

NASA expects the use of DT technology to halve aircraft maintenance costs and extend service life to 10 times its current level by 2035 [44]. The application of DT in aerospace has been widely concerned at home and abroad.

In the aviation system, 6 sigma methods have been used to assess spacecraft quality and solution processes have been established for weaknesses in aviation issues, including identification, analysis, improvement, evaluation, and sharing of five phases [21]. The digital model of the spacecraft is constructed by the DT, and the 6 sigma process is simulated in the digital model to ensure the quality of the space engineering. The computer products used in aerospace are designed to optimize the production management and quality management process of aerospace equipment parts, and can operate reliably and efficiently under simulated conditions. The rapid development of aerospace computer products has an important guarantee for improving the risk management capabilities of spacecraft and ensuring the smooth execution of space missions [81]. The manufacture of aeronautical parts requires rigorous monitoring to ensure the quality of the parts. Real-time monitoring, analysis and control of the machining process are indispensable for optimizing aerospace parts manufacturing and machining strategies. Digital Twinss provide an efficient technical means for the modeling and analysis process of aerospace equipment. DT technology can predict, perceive, control and optimize various state changes in the aerospace equipment manufacturing process by integrating multi-dimensional processing data, such as changes in geometry, material properties and processing parameters. Shimin [40] creatively proposed that the DT system is similar to the biological imitation phenomenon of nature, combined with the idea of biological imitation, made a digital model with adaptive characteristics, and in the manufacture of rocket cabin tail air rudder has been practical application. Albrecht [31] also proposes a way to create a DT model based on planning and processing data. The DT are used in the processing of space-going parts, identify problems directly during the processing process, and propose targeted measures to ensure the quality of parts and optimize the processing process. The application has been applied to the milling steps of aerospace equipment.

**Fig. 9:**
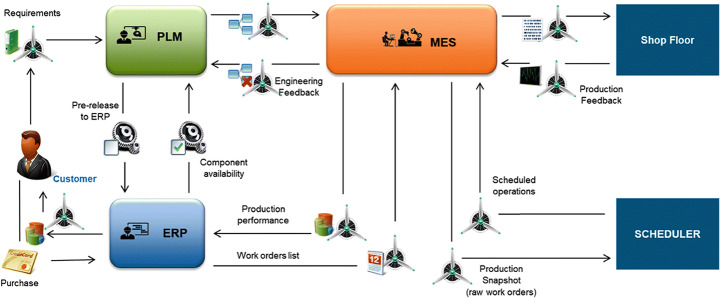
PLM and CLM combined architecture [57]

In addition to improving the spacecraft’s production process, DT can also go deep into the life cycle of space equipment, monitor the operational status of space equipment in real time, facilitate the maintenance of equipment, and improve its design structure. Glaessgen and Stargel [28] proposed a simulation model based on Digital Twinss to monitor equipment abnormalities, to maintain the health of space equipment, and to maintain historical data. Reifsnider and Majumdar [50] proposed a DT of an aircraft, by analyzing data from the aircraft, predicting the early occurrence of microcracks in the equipment. Tuegel [68] developed DT based on virtual health sensors using FEM and Montecarlo simulation technology and predicted maintenance requirements with damage and durability emulator simulation software. They suggest that ADT uses Bayesus updates to reduce maintenance costs by reducing uncertainty and increasing service information. In the EC-funded Artemis CRYSTAL project, the authors applied DT to the aeronautical case study of the CRYSTAL research project, designed an anti-ice system for regional turboprop aircraft for the CRYSTAL project, simulated and predicted the anti-ice effect of the system, and further improved the system based on the collected data [6]. In the area of digital modeling, Ríos [52] proposed a DT based on Dassault V6 simulation system for aircraft design. Salinger [57] proposed a test platform focusing on aerospace equipment hardware for the development of a dynamic data-driven platform. The purpose of this platform is to realize an unmanned aerial vehicle with self-awareness. DT is applied to analyze dynamic data (Fig. 9).

With DT applied to architecture in the aerospace industry, the AirGreen 2 project proposes the Product Lifecycle Management (PLM) and Manufacturing Operation Man-Agement(MOM) architectures for the aerospace industry. Incorporating this system into enterprise production can improve order management efficiency and create automated working methods to coordinate supply chain processes and optimize material synchronization [18]. Use PLM to collect production requirements, make a production plan, transmit it to the MES, and finally execute it in the factory. At the same time, MES continuously receives feedback from the factory and sends it back to PLM to optimize the production plan. ERP is used for resource planning and at the same time provides a basis for production decision-making.

DT allow real data to be applied to simulation systems, and have very broad prospects in the field of aerospace [58]. At present, although DT have made some progress in aerospace, because aerospace-related technologies are too complex, and the safety and real-time nature of technology is very demanding. Judging from the literature currently available, the application of DT in aerospace is still in its infancy. The application of DT in the aerospace field still faces some challenges:

1. The aerospace industry is a comprehensive field involving many fields, which improves the difficulty of realizing DT, and the model structure definition of DT technology framework and DT is not yet mature, how to reason knowledge based on DT, discover to achieve intelligence. The ultimate goal of digitalization has yet to be studied;
2. In the key technologies of DT, fault diagnosis based on big data, dynamic modeling of complex systems under uncertainty, online real-time analysis and computational mathematical methods, lightweight resistance to extreme conditions in aerospace environments, distributed sensing monitoring technology are the leading issues in current research;
3. At the level of DT tools, it is still necessary to develop and perfect the development and operation integration platform of autonomous DT.

## The application of DT in the business world

With the development of digital technology, completely channel sales has become a business norm in the retail industry. Buyers can choose the shopping channel that best suits them. Retailers take the business model that best suits the channel, depending on the characteristics of the channel [46]. For example, a virtual online display cabinet is available online, allowing customers to understand as much as possible about the product without touching it. Or display the product information online and mail it to the customer. The last form we call online shopping, which is also the hottest business model in recent years. Both online and offline sales channels are the two most mainstream sales models.

### Offline sales

For offline displays of goods, the widespread use of digital technology has changed the way we interact with goods over the past more than ten years, and the interaction between people and information has become more and more close. Back in 2015, Antonella deployed a smart screen interaction device in a mobile store that allows users to connect to the large screen while they waiting [51]. Antonella then proposed a new interactive system for smart retail environments that leverages technologies already familiar to shoppers (smartphones, smartwatches, touch screens, etc.) to provide users with a new way to interact [53]. Although these methods improve the efficiency of user-commodity interaction to some extent, they lack an immersive experience. We also need a new multimedia technology to enhance the interaction between users and goods. Recently, a paper survey in punthira [13] found that digital technology is widely used in business, with the highest heat including DT technology.

DT can construct a virtual mirror image of the product, so that customers can “touch” the product without opening the product packaging. For valuable products, it allows users to experience the product without touching the actual product, which can greatly increase the user’s interest in product purchases. And to a certain extent to prevent users from being dissatisfied with the goods, thereby requesting a return of the situation. For the business, it also prevents the goods from being damaged by display.

### Online sales

The Covid-19 has changed the way people live. The arrival of the epidemic has forced people to stay at home, leading many to change their way of life. People now work from home and study from home. People who are used to eating in restaurants start learning to order takeaways at home. The most changed is online shopping, so many people have learned to shop online. The share of online retailing in the United States, from 4.5% to 2011 to 10% in 2020, took nine years. According to the U.S.Census Bureau,the share of online retailing has grown to 16% in six months this year because of the Covid-19. Inevitably as the number of online stores increases, the competition among the major stores is becoming more and more intense. In order to increase turnover, shopkeepers need new ways to attract users. Meredith [43] surveyed 200 people and analyzed their shopping history and found that public psychology had a significant impact on users’ purchases. Therefore, increasing store sales will be the goal pursued by the major online stores. There are many commonly used schemes for merchants, such as adding more compelling content, offering limited-time offers, and providing users with personalized recommendations. Kawabe [34] designed an adaptive system that automatically adjusts to sales conditions. In this system, the user’s browsing and consumption records are obtained to obtain the needs of consumers, and a personalized product recommendation list is automatically formulated.

On the one hand, these promotional methods can help customers find the goods they need, which in turn increases the turnover of the store. On the other hand, for those who have just started shopping online, the biggest concern is whether the products are in line with their own desires. This is also a big disadvantage of online shopping compared to offline shopping. DT technology can dispel customers’ concerns. By creating virtual spaces online, generate DT of users and products, allowing users to experience goods in virtual spaces. At present, there has been a certain breakthrough in the online commodity experience. Kim’s [35] digital fit technology designs virtual human bodies (VHBs). With the improvement of this technology, consumers can try on clothes in the online virtual store, make up for the lack of online shopping. Alibaba also introduced the Buy Plus concept in 2016, which is to achieve a full range of shopping experiences through virtual reality devices [25].

**Fig. 10:**
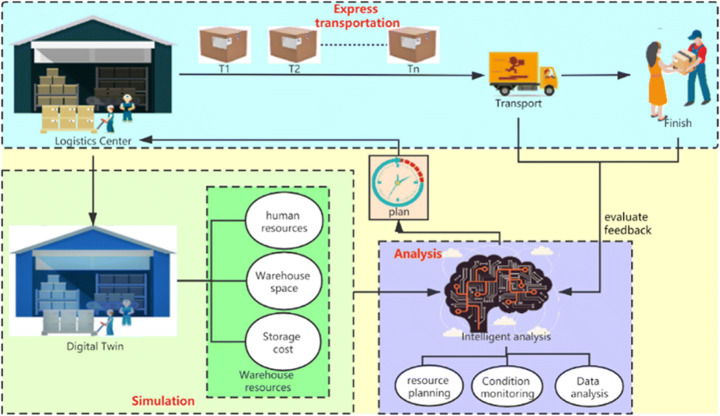
DES applied in DT

Online shopping also has an important link —— logistics. This is also one of the most concerned aspects of customers. In order to ensure that the goods can reach the hands of buyers smoothly, the physical management of goods is also a very important link. Warehouse as the starting point of logistics, but also the starting point of logistics management. For better management of warehouses, the concept of discrete event simulation Discrete Event Simulation (DES) has been proposed. DES is the concept of a systematic process, with each step looping together. Virtualize the entire process with DT for simulation, predicting system performance, system interactions, and decision improvements [1]. DT integrated with DES will be the best solution for logistics management. Ability to plan efficient warehouses, better manage warehouses, and make decisions. At the same time improve the efficiency of the logistics industry and service quality. In the future, it will even be available to generate DT for each commodity, which can be tracked in real time from the process of production, storage, transportation, etc. to ensure the safety of the commodity. The DT can generate a digital mirror mapping of the logistics center, perform dynamic simulation. Through intelligent analysis, the warehouse’s human resources, warehouse space, and storage costs are planned, and the changed plan is fed back to the actual warehouse. When the logistics is completed, the feedback of users and couriers will be summarized and analyzed to provide a basis for subsequent decision-making(Fig. 10).

## Prospect

Due to the rapid development of science and technology, DT are still a concept in constant development. At present, there are still many problems to be solved in the promotion of DT to practical applications. Such as model simulation, data security and other key technology. We should also strengthen the construction of an open and communication platform for the DT production industry, promote the integration of DT with other technologies, so as to make DT better able to use real life and work. The most critical and difficult thing to achieve is to develop international common standards for DT for all industries. Only after solving these problems can the DT be popularized in real production and the process of economic and social digitization can be accelerated.

1. At present, industrial production has begun to show its strength. Schuler, Siemens and other large companies have used DT in actual production, and achieved some results. Although DT technology is not yet mature, but with the deepening of research, the benefits of DT for industrial production is predictable. In the process of development, the industrial industry makes full use of digital technology, which can effectively improve production efficiency and optimize product quality, while at the same time making the product have good performance. This day and age, digital production process and digital design have been widely used in industrial production. Digital technology can speed up the technological innovation of industrial industry, optimize the traditional industrial production model to the maximum extent, and make the industrial production system become more intelligent. At present, the industrial industry in the Internet technology under the generalization has gradually formed the industrial intelligence, DT is the key to the development of industrial intelligence. With the advent of the 5G era, the industrial Internet has also been widely used, so that industrial enterprises around the world will pay attention to and apply DT technology. DT technology can help people build new perspectives from a digital-driven perspective, re-examine flaws in traditional enterprise models, and innovate space. Especially after using DT technology, the original simulation technology and big data, IOT, artificial intelligence and so on can be deeply integrated. Applying DT to industrial production can revolutionize industrial product design, production processes, equipment maintenance, etc. The current development trend of DT in industry:
  1. Industrial production equipment intelligence. In order to realize the intelligent development of industry, we should first ensure the intelligence of industrial production equipment, that is, to ensure that the equipment can run normally. Build a production facility’s DT from a DT. Monitor equipment status in real time and predict possible failures to reduce maintenance costs.
  2. Intelligent manufacturing process. Intelligent production is to ensure that the production process is safe and reliable, as far as possible to reduce labor costs. In the production should make full use of intelligent systems and equipment, to achieve equipment operation automation and unmanned operation. In brief, it is to use DT to monitor the production process, ensure the normal operation of production, and ensure product quality.
  3. Product quality inspection intelligence. At present, for the product quality inspection process needs to consume a lot of human and material resources. DT simplify the quality inspection process. DT combine with AI to discover products with quality problems in a timely manner. And according to the different problems of the product classification, analysis of the cause of the problem, and then to ensure product quality.
2. DT can greatly improve modern medical care and make many contributions to the protection of human health. Specifically, medical care can be broadly divided into two stages: treatment and health care. In the treatment of diseases, DT can build DT of human organs. This is also the primary medical application of DT. By constructing a patient’s organs, doctors can more intuitively analyze surgical options. In the future, artificial organs may even be constructed from DT to replace diseased organs. The second is personalized medicine. To be honest, that’s what I think DT need to develop aggressively in patient care. Personalized medicine can accurately analyze the patient’s physical condition and develop personalized treatment plan. This reduces the waste of medical resources and the side effects of the drug. Doctors can also analyze patients online based on models obtained from DT. In addition, drug research and development in virtual human bodies, combined with virtual simulations at the molecular cell level to conduct virtual and clinical trials of drugs, can greatly reduce the drug development cycle. In addition, health care is also a very important aspect. Now people pay more and more attention to health. DT enable the digitization of a person’s body, behavior, and consciousness. Human DT contribute to health management and information tracking and can play an irreplaceable role in responding to emergencies. As a consequence, I envision the best level of human medicine in the future, which is to build a human DT from birth, as a life-long archive, record a person’s life.
3. In smart cities, DT can simulate urban construction and planning, population change, traffic operations, public security management, cultural tourism, health care and education services. DT is an effective technical means to realize smart cities, with the help of DT cities, can improve the quality and level of urban planning, promote urban design and construction, assist urban management and operation, so that urban life and environment better. The DT of the future will enable citizens to participate in the governance of cities. At the same time, in the event of an outbreak, the DT can also simulate the spread of the epidemic and the movement of people and supply and demand of goods in the traffic control of real cities. Currently, the construction difficulty of smart city is mainly in the aspect of urban simulation. How to create the dynamic model of the city completely and accurately is one of the important problems that need to be solved in the future.
4. DT are widely used in the field of spaceflight. To be specific, there are many applications in the simulation flight, flight detection and fault analysis of spacecraft. It greatly improves the success rate of spacecraft launch and reduces the cost of launching space vehicles. In the future, DTT could be used in the field of satellites. After the satellite enters orbit, its health monitoring and maintenance is very complex. The introduction of DT technology into the full life cycle of satellites enables real-time monitoring of space satellites. And with the simulation of twin models, satellites can be assisted in experiments by ground assistance. As satellite technology matures, DT can then be used to manage the space station.
5. In the business sector, the main objective of DT is to increase sales. Merchants use DT to create diamond rings, crystals, watches and other valuable goods, so that customers do not need to touch the goods to try out the goods. In addition, some online merchants are currently building digital models of stores online. Customers can experience goods more intuitively and improve their shopping experience. In the future, users will be provided with a DT of the store directly, allowing them to experience offline shopping from home. With the continuous development of online shopping, the future logistics requirements will be more and more stringent. Using a DT-based three-dimensional logistics center design, according to the needs of the transport of goods, to achieve personalized customization of warehouse. Remote operation service platform can remotely dispatch and process warehouse information, improve the efficiency of warehouse operation. Shared three-dimensional warehouse can maximize the use of resources, save resources, reduce costs.

## Conclusion

This paper first introduces the practical application of DT in various fields, then expounds the current situation and needs in various fields, and finally explains the development focus of DT in various fields.

In the field of industrial production, DT have great advantages in industrial manufacturing, model construction, plant management, etc. In particular, DT have a natural advantage when it comes to equipment maintenance. In the medical field, DT have many applications in disease treatment, health management and exercise planning. It can be treated by paramedics and can even predict disease. The most prominent contribution of DT in the medical field is the ability to personalize care for patients. DT are currently primarily used in education to increase the diversity of teaching. In the future, DT could serve as “private teachers” to help students develop personal learning plans. DT are the key technologies for building smart cities and can play a very important role in urban planning, citizen management, resource management and so on. Most of the current cases of smart cities are in their infancy, and it is expected that DT will play a greater role in the construction of smart cities. The space field is the starting point of the word twin, and it is also the earliest field to realize the application. Using DT can significantly reduce space costs. Many of the technologies implemented in the space industry will also be used in enterprises or private sectors when they are relatively mature. The development of DT in the space field is also conducive to technological advances in other fields. Business is a very big problem, the current DT applications in business are mainly divided into online and offline two kinds. The fundamental purpose of a merchant’s use of DT is to increase store sales. At the same time, DT also have some applications in the simulation of discrete events in logistics.

Digital transformation is the only way for the future development of the world economy. From an academic point of view, the use of DT is also increasing. In the future, for DT to enter our lives, DT applications must be accurate to win the trust and trust of users, and must be robust enough to allow users to use them normally [56]. It is hoped that the DT of the future will enter our lives and accelerate the digitization of the world.
